# Renal Impairment and Prognosis of Patients with Atrial Fibrillation Undergoing Coronary Intervention - The AFCAS Trial

**DOI:** 10.1371/journal.pone.0128492

**Published:** 2015-06-01

**Authors:** Heli M. Lahtela, Tuomas O. Kiviniemi, Marja K. Puurunen, Axel Schlitt, Andrea Rubboli, Antti Ylitalo, José Valencia, Gregory Y. H. Lip, K. E. Juhani Airaksinen

**Affiliations:** 1 Heart Center, Turku University Hospital and University of Turku, Turku, Finland; 2 Hemostasis laboratory, Finnish Red Cross Blood Service, Helsinki, Finland; 3 Medical Faculty, Martin Luther University Halle-Wittenberg and Paracelsus-Harz-Clinic Bad Suderode, Halle, Germany; 4 Division of Cardiology, Laboratory of Interventional Cardiology, Ospedale Maggiore, Bologna, Italy; 5 Department of Internal Medicine, Lapland Central Hospital, Rovaniemi, Finland; Medical Research Center, University of Oulu, Oulu, Finland; 6 Department of Cardiology, General Hospital University of Alicante, Alicante, Spain; 7 University of Birmingham Centre for Cardiovascular Sciences, City Hospital, Birmingham, United Kingdom; KRH Robert Koch Klinikum Gehrden, GERMANY

## Abstract

**Background:**

Renal impairment is a well-known risk factor for cardiovascular complications, but the effect of different stages of renal impairment on thrombotic/thromboembolic and bleeding complications in patients with atrial fibrillation (AF) undergoing percutaneous coronary intervention (PCI) remains largely unknown. We sought to evaluate the incidence and clinical impact of four stages of renal impairment in patients with AF undergoing PCI.

**Methods:**

We assessed renal function by estimated glomerular filtration rate (eGFR) and outcomes in 781 AF patients undergoing PCI by using the data from a prospective European multicenter registry. End-points included all-cause mortality, major adverse cardiac and cerebrovascular events (MACCE) and bleeding events at 12 months.

**Results:**

A total of 195 (25%) patients had normal renal function (eGFR ≥90 mL/min), 290 (37%) mild renal impairment (eGFR 60-89), 263 (34%) moderate renal impairment (eGFR 30–59) and 33 (4%) severe renal impairment (eGFR <30). Degree of renal impairment remained an independent predictor of mortality and MACCE in an adjusted a Cox regression model. Even patients with mild renal impairment had a higher risk of all-cause mortality (HR 2.25, 95%CI 1.02-4.98, p=0.04) and borderline risk for MACCE (HR 1.56, 95%CI 0.98- 2.50, p=0.06) compared to those with normal renal function.

**Conclusions:**

Renal impairment is common in patients with AF undergoing PCI and even mild renal impairment has an adverse prognostic effect in these patients requiring multiple antithrombotic medications.

## Introduction

Chronic kidney disease (CKD) has become an increasing problem affecting up to 16% of people globally [1]. Several studies have established that in the general population moderate and severe CKD is associated with an increased risk for cardiovascular morbidity and mortality, and increasing prevalence and incidence of atrial fibrillation (AF) [2–9]. Traditionally CKD has been defined as kidney damage or estimated glomerular filtration rate (eGFR) lower than 60 mL/min per 1.73 m^2^ of body-surface area. With this definition CKD has also been associated with an increased risk of ischemic as well as bleeding complications after percutaneous coronary intervention (PCI) in various patient groups [10–14]. National Kidney Foundation guidelines define, however, 5 stages of CKD based on eGFR and even the early stage of disease (eGFR 60-89mL/min) seems to increase cardiovascular risk irrespective of levels of traditional cardiovascular disease risk factors [15, 16].

About 5 to 8% of patients referred for PCI have an indication for long-term anticoagulation; mainly due to AF [17, 18]. In patients with AF, CKD is associated with an increased risk of stroke and/or systemic thromboembolism and bleeding complications [19, 20]. The prevalence of CKD in patients with AF undergoing PCI remains largely unknown. Moreover, the effect of renal impairment on the outcomes of these patients requiring multiple antithrombotic medications is not known. In this pre-specified analysis, our aim was to assess the impact of renal impairment on outcome of PCI in patients with AF in the large multicenter prospective AFCAS (Atrial Fibrillation undergoing Coronary Artery Stenting) registry with a special emphasis on the different stages of CKD.

## Methods

### Registry data sources

AFCAS is an observational, multicenter, prospective registry including patients with AF who are referred for PCI (Clinicaltrials.gov identifier NCT00596570)[21–24]. Between October 2008 and February 2010, 963 consecutive patients with AF undergoing PCI were included the AFCAS registry at 17 centers in 5 European countries: Finland, Germany, Italy, Spain, and the UK. Because of the observational design of the study, the only exclusion criteria were unwillingness/inability to participate in the study or to give written informed consent.

At each participating center, patients were treated according to local policies, and were followed up for 12 months (phone call or visit at 3, 6 and 12 months). The study protocol were approved by the Ethical Committee of the coordinating center (Hospital District of South-Western Finland, Turku, Finland) and participating centers, and written informed consent was obtained from every patient. The study complied with the Declaration of Helsinki.

Coronary angiography and PCI were performed using either radial or femoral approach for arterial access and hemostasis was obtained according to local practice. Lesions were treated according to contemporary interventional techniques. Low-molecular weight heparins, unfractionated heparin, bivalirudin and glycoprotein IIb/IIIa inhibitors were administered entirely at the operator’s discretion. Medication at discharge was at the treating physician’s discretion.

### Study population

Of the 975 patients included in the AFCAS registry, the data to calculate pre-PCI eGFR were available for 781 (80%) patients. The patients were divided into four groups according to their baseline eGFR levels: ≥90 ml/min/1.73 m^2^ as normal renal function; 60 to 89 ml/min/1.73 m^2^ as mild renal impairment; 30 to 59 ml/min/1.73 m^2^ as moderate renal impairment; and <30 ml/min/1.73 m^2^ as severe renal impairment. There were only 7 patients with eGFR <15ml/min, and they were included in group with eGFR <30ml/min. The Cockcroft—Gault formula was used in the calculation of eGFR [16].

### End-point definitions and clinical follow-up

The endpoints were 1) all-cause mortality; 2) a composite of major cardiac and cerebrovascular events (MACCE) including all-cause mortality, myocardial infarction, non-elective repeat revascularization (PCI or coronary bypass surgery of either target vessel or target lesion or any other acutely occluded coronary artery but excluding elective revascularization of other coronary arteries), stent thrombosis, transient ischemic attack, stroke, other arterial embolism; and 3) bleeding during 12 months follow-up period.

Periprocedural myocardial infarction was not routinely screened, but if suspected, a troponin level > 3x normal 99th percentile level was required for the diagnosis. For the diagnosis of myocardial re-infarction, a new rise > 50% above the baseline injury marker level was required. Stent thrombosis was defined according to the Academic Research Consortium (ARC) Classification as definite and probable [25]. TIA was defined as a focal transient (<24h) neurological deficit adjudicated by a neurologist and stroke as a permanent focal neurological deficit adjudicated by a neurologist and confirmed by computed tomography or magnetic resonance imaging. Systemic embolism was defined as signs/symptoms of peripheral ischemia associated with a positive imaging test.

Bleeding complications were defined according to the ARC bleeding criteria (BARC) as any bleeding complications (BARC 1–5), minor (BARC 2), and major (BARC 3a, 3b, 3c and 5) [26]. Acute kidney injury was defined with as >26.5 μmol/l increase of creatinine, using modified definition by Kidney Disease: Improving Global Outcomes (KDIGO) Acute Kidney Injury Work Group. The exact time points for blood samples were not defined in the protocol, but these were the routine PCI-samples drawn before the procedure and on the 1^st^ post-PCI morning.

### Statistical analysis

Continuous variables with normal distribution are reported as mean ± standard deviation (SD), skewed variables as median and interquartile range (IQR), tested by ANOVA (Bonferroni) for normally distributed data and by non-parametric (Kruskal—Wallis one-way analysis of variance) tests otherwise. Categorical variables are presented as percentage and Chi-square test and Fisher’s exact test were used where appropriate. P value < 0.05 was considered statistically significant. Kaplan-Meier analyses were used to construct survival plots of time to death, MACCE and bleeding events after PCI. Multivariate adjusted analysis of predictors of mortality; MACCE and bleeding events were done using Cox regression models. Multivariate analysis included variables with significant baseline or periprocedural differences. Patients with normal eGFR (>90mL/min) was used as a reference category. All computations were carried out with SPSS software (V20.0, SPSS Inc., Chicago, Illinois, USA).

## Results

### Baseline and procedural characteristics

Pre-PCI creatinine was available in 781 patients (69.7% men; median age 74 years, range 45–92). A total of 195 (25%) patients had normal eGFR (≥90 mL/min), 290 (37%) mild renal impairment (eGFR 60–89), 263 (34%) moderate renal impairment (eGFR 30–59) and 33 (4%) severe renal impairment (eGFR <30). Baseline characteristics of each eGFR group are shown in Table 1. Patients with severe renal impairment were older, more often female and more often presented with acute coronary syndrome as an indication for PCI (Tables 1 and 2). There were no significant differences in the use of drug-eluting stents, access site, perioperative medications or other procedural characteristics between the groups. (Tables 2 and 3) The use of evidence-based cardiac medications was comparable between the study groups at discharge, excluding lipid-lowering agents which were less often used in patients with severe renal impairment (Table 3). The hospital stay was significantly longer in patients with moderate and severe renal impairment.

**Table 1:** Baseline characteristics of the study population.

All	eGFR ≥90	eGFR 60–89	eGFR 30–59	eGFR <30
N = 781	n = 195	n = 290	n = 263	n = 33
Male	164 (84.1)	209 (72.1)**	151 (57.4)**	20 (60.6)**
Age (year)	65.5±7.7	73.4±6.7**	77.7±5.4**	77.0±6.7**
eGFR prePCI	120±28	74±9**	47±8**	21±6**
CHA_2_DS_2_-VASC				
mean ±SD	3.5±1.3	4.4±1.4**	5.0±1.3**	5.0±1.5**
CHA_2_DS_2_-VASC ≥2	188 (96.4)	288 (99.3)*	262 (99.6)*	33 (100)
HAS-BLED				
median (IQR)	3.0 (1.0)	3.0 (0.25)**	3.0 (1.0)**	4.0 (1.0)**
mean ±SD	2.58 ± 0.73	2.92 ± 0.72	3.17 ±0.62	3.76 ± 0.79
Ejection fraction (%)	51 ± 13	50 ± 14	48 ± 14*	48 ±12
Diabetes	72 (36.9)	100 (34.5)	90 (34.2)	18 (54.5)
Hypertension	167 (85.6)	228 (78.6)	229 (87.1)	30 (90.9)
Hypercholesterolemia	129 (66.2)	208 (71.7)	166 (63.1)	22 (66.7)
Smoking	40 (20.5)	19 (6.6)**	17 (6.5)**	4 (12.1)
Medical history				
Myocardial infarction	39 (20.0)	84 (29.0)*	65 (24.7)	13 (39.4)*
PCI	27 (13.8)	56 (19.3)	44 (16.7)	6 (18.2)
CABG	17 (8.7)	53 (18.3)**	40 (15.2)*	5 (15.2)
Heart failure	30 (15.4)	67 (23.1)*	70 (26.6)**	10 (30.3)*
Stroke/TIA	27 (13.8)	47 (16.2)	51 (19.4)	4 (12.1)
Prior hemorrhage	6 (3.1)	14 (4.9)	12 (4.6)	1 (3.0)
Number of patients per country				
Finland	125 (29.6)	171 (40.5)	119 (28.2)	7 (1.7)
Germany	57 (22.5)	86 (34.0)	90 (35.6)	20 (7.9)
Italy	4 (6.7)	20 (33.3)	31 (51.7)	5 (8.3)
Spain	9 (19.6)	13 (28.3)	23 (50.0)	1 (2.2)

P-value < 0.05 = *, p value < 0.01 = **, all values are compared with eGFR ≥90 ml/min/1.73 m² group. Data are reported as number and percentage or mean ± SD.; eGFR = estimated glomerular filtration rate; CHA₂ DS₂ VASC = C ongestive heart failure, H ypertension, A ge ≥75 years, D iabetes, prior S troke/transient ischaemic attack/systemic embolism, associated V ascular disease, A ge 65–74 years, and female S ex category; HAS-BLED = Hypertension, Abnormal liver or kidney function, prior Stroke, Bleeding history or predisposition, Labile INR, Elderly, and concomitant Drugs; PCI = percutaneous coronary intervention; CABG = coronary artery bypass graft surgery; TIA = transient ischemic attack.

**Table 2:** Procedural characteristics.

All	eGFR ≥90	eGFR 60–89	eGFR 30–59	eGFR <30
N = 781	n = 195	n = 290	n = 263	n = 33
Indication for PCI				
Stable angina	95 (48.7)	138 (47.6)	108 (41.1)	8 (24.2)*
ACS	100 (51.3)	152 (52.4)	155 (58.9)	25 (75.8)*
STEMI	25 (12.8)	26 (9.0)	36 (13.7)	4 (12.1)
NSTEMI	39 (20.0)	69 (23.8)	69 (26.2)	14 (42.4)**
Lesions treated per patient	1.1 ± 0.4	1.2 ± 0.4	1.2 ± 0.4	1.3 ± 0.5
Drug-eluting stents	49 (25.1)	68 (23.4)	53 (20.2)	7 (21.2)
Total stent length (mm)	25.8 ± 16.1	24.5 ± 5.4	24.8 ± 17.1	31.7 ± 19.8
Procedural success	192 (98.5)	279 (96.2)	257 (97.7)	32 (97.0)
Radial access	48 (24.6)	71 (24.5)	75 (28.5)	9 (27.3)
Access site complications	18 (9.2)	24 (8.3)	20 (7.6)	3 (9.7)
Length of hospitalization (days)	4.2 ± 5.2	4.2 ± 6.2	5.9±8.2**	8.8±6.7**

P-value < 0.05 = *, p value < 0.01 = **, all values are compared with eGFR ≥90mL/min group. Data are reported as number and percentage or mean ± SD; eGFR = estimated glomerular filtration rate; PCI = percutaneous coronary intervention; ACS = acute coronary syndrome; STEMI = ST-elevation myocardial infarction; NSTEMI = non ST-elevation myocardial infarction

**Table 3:** Cardiac and antithrombotic medication at discharge.

All	eGFR ≥90	eGFR 60–90	eGFR 30–60	eGFR <30
N = 781	n = 195	n = 290	n = 263	n = 33
Periprocedural INR	1.9 ± 0.7	2.0 ± 0.7	1.9 ± 0.7	1.9 ± 0.8
Glycoprotein IIb/IIIa inhibitors	49 (25.1)	53 (18.3)	58 (22.1)	9 (27.3)
VKA + Clopidogrel + Aspirin	151 (77.4)	211 (72.8)	179 (68.1)*	21 (63.6)
VKA + Clopidogrel	8 (4.1)	23 (7.9)	25 (9.5)*	2 (6.1)
Clopidogrel + Aspirin	32 (16.4)	50 (17.2)	55 (20.9)	10 (30.3)
Clopidogrel duration				
month	6.0±4.8	5.5±4.6	5.9±4.8	6.2±4.8
median (min-max)	4 (0.5–12)	3 (0.25–12)	3 (0–12)	3.5 (0.4–12)
Beta blockers	174 (89.2)	260 (89.7)	219 (83.3)	29 (87.9)
Lipid-lowering agents	175 (89.7)	258 (89.0)	218 (82.9)	25 (75.8)*
ACEi / ARB	160 (82.9)	227 (79.6)	212(83.5)	25 (80.6)

P-value < 0.05 = *, p value < 0.01 = **, all values are compared with eGFR ≥90mL/min group. Data are reported as number and percentage or mean ± SD; eGFR estimated glomerular filtration rate; VKA, vitamin K antagonist; INR, international normalized ratio; ACEi, angiotensin-converting enzyme inhibitors; ARB, angiotensin receptor blockers

In the overall cohort, triple therapy consisting of vitamin K antagonist, clopidogrel and aspirin was used in 72% of the patients after PCI. The use of triple therapy tended to decrease in the study groups along with decreasing renal function (77.4 vs 72.8 vs 68.1 vs 63.6%). The duration of clopidogrel use was comparable in all eGFR groups (Table 3).

### Outcomes

**Fig 1:**
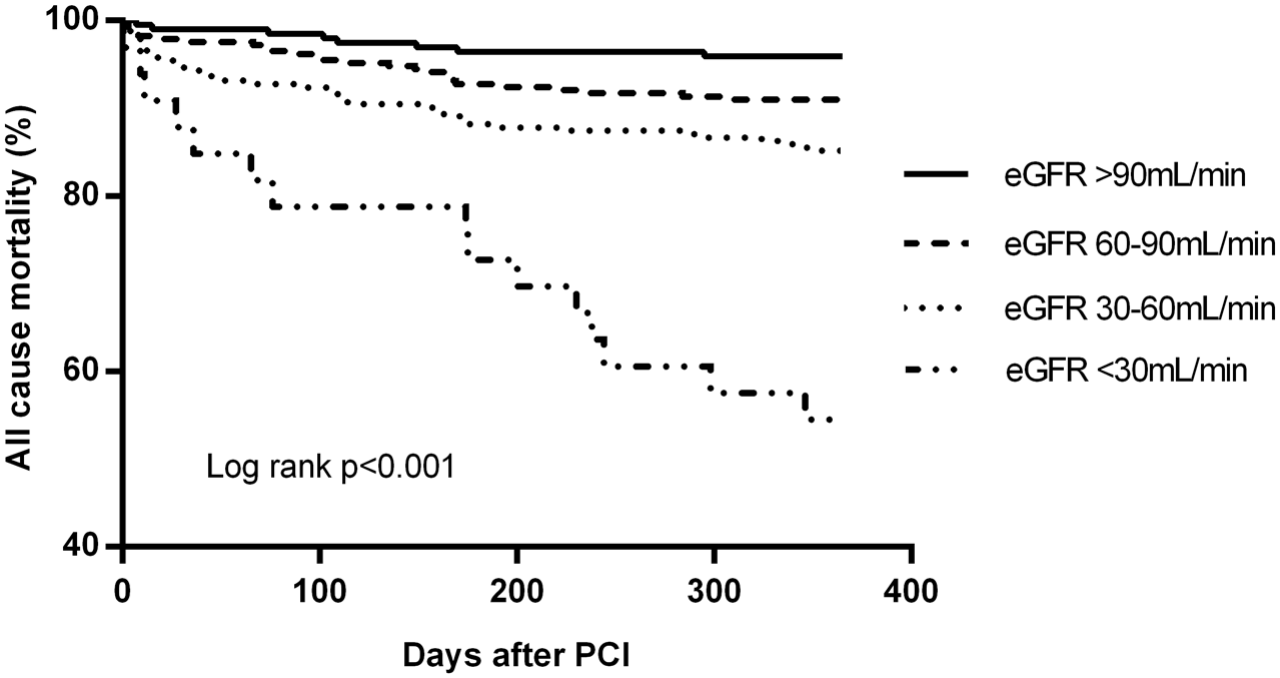
Freedom from all-cause mortality according to eGFR groups at 12-month follow-up.

**Fig 2:**
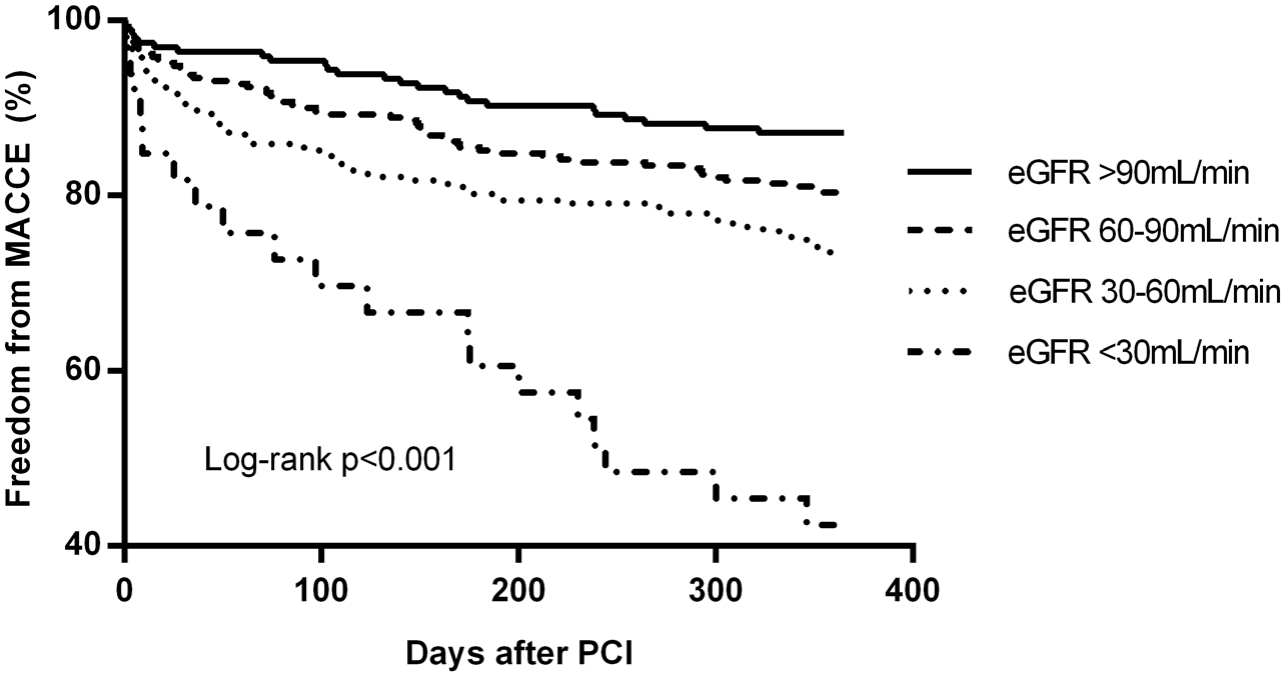
Freedom from MACCE according to eGFR groups at 12-month follow-up.

**Fig 3:**
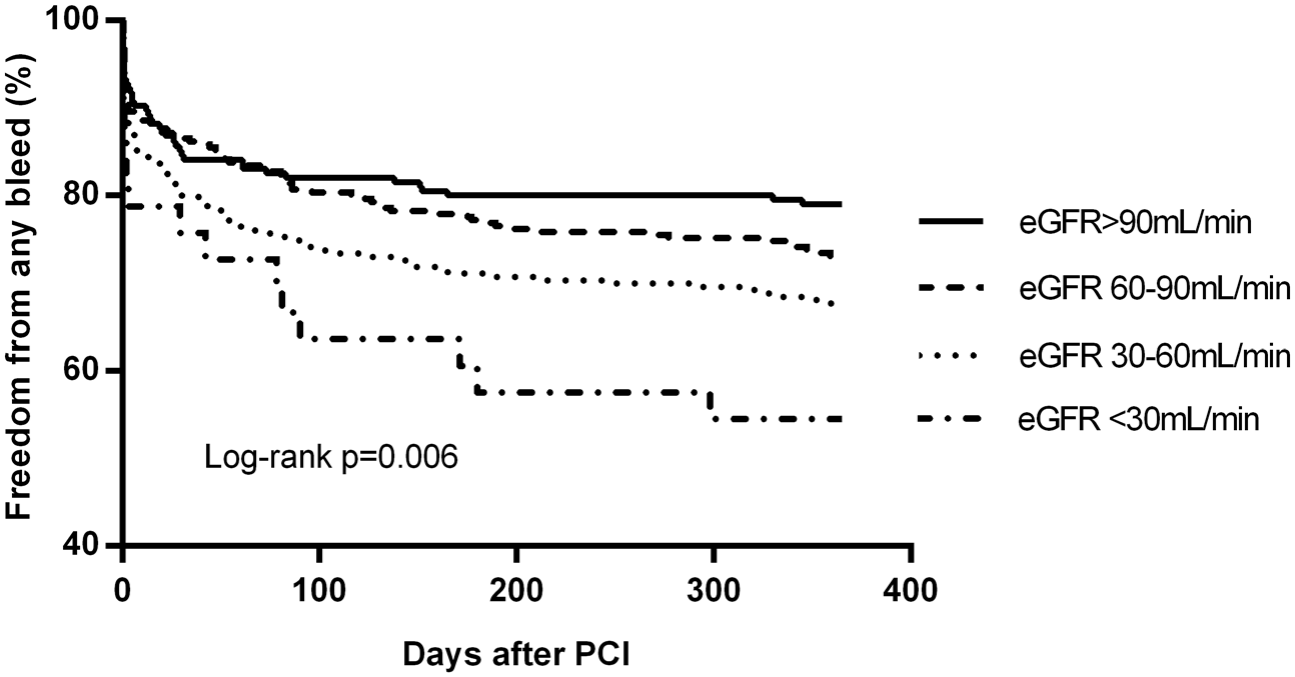
Freedom from any bleeding event according to eGFR groups at 12-month follow-up.

Outcome events at 12 months follow-up are presented in Table 4. Figs 1–3 present the Kaplan-Meier estimates for all-cause mortality, MACCE and all bleeding events, respectively. Overall, the rates of stroke and stent thrombosis were low. Renal impairment remained an independent predictor of mortality and MACCE in a multivariate Cox regression model adjusted for age (as a continous variable) and sex (Model A) or age, sex and acute coronary syndrome (Model B) or age, sex, acute coronary syndrome, previous myocardial infarction, congestive heart failure and center (Model C) (Table 5). The patients with mild renal impairment had a higher risk of all-cause mortality (HR 2.25, 95%CI 1.02–4.98, p = 0.04) and borderline risk for MACCE (HR 1.56, 95%CI 0.98–2.50, p = 0.06) compared to those with normal eGFR. Patients with moderate renal impairment had a higher risk of all-cause mortality (HR 3.77 95%Cl 1.76–8.09, p = 0.001) and MACCE (HR 1.99 95%CI 1.25–3.17, p = 0.004) compared to those with normal eGFR. Moreover, they had slightly increased risk for mortality compared to patients with mild renal impairment (HR 1.67 (1.02–2.76), p = 0.04).

**Table 4:** Outcome events at 12-month follow-up.

All	eGFR ≥90	eGFR 60–90	eGFR 30–60	eGFR <30
N = 781	n = 195	n = 290	n = 263	n = 33
MACCE				
12-months	25 (12.8)	58 (20.0)*	70 (26.6)**	19 (57.6)**
30 days	7 (3.6)	17 (5.9)	25 (9.5)*	6 (18.2)**
In-hospital	5 (2.6)	11 (3.8)	14 (5.3)	3 (9.1)
DEATH				
12-months	8 (4.1)	26 (9.0)*	39 (14.8)**	15 (45.5)**
30 days	2 (1.0)	10 (3.4)	17 (6.5)**	5 (15.2)**
In-hospital death	2 (1.0)	5 (1.7)	8 (3.0)	2 (6.1)
Myocardial infarction				
12-months	4 (2.1)	10 (3.4)	18 (6.8)*	5 (15.2)**
in-hospital	0	0	3 (1.1)	1 (3.0)
Re-revascularization	13 (6.7)	28 (9.7)	22 (8.4)	4 (12.1)
Stent thrombosis	1 (0.5)	5 (1.7)	6 (2.3)	0
Stroke/TIA	4 (2.1)	11 (3.8)	6 (2.3)	2 (6.1)
All thromboembolism	4 (2.1)	11 (3.8)	10 (3.8)	3 (9.1)
All bleeding (BARC 1–5)				
12 months	41 (21.0)	80 (27.6)	86 (32.7)**	15 (45.5)**
30 days	30 (15.4)	53 (20.2)	39 (13.4)	8 (24.2)
In-hospital	20 (10.3)	29 (10.0)	34 (13.0)	7 (21.2)
BARC >2				
12 months	17 (8.7)	27 (9.3)	33 (12.5)	5 (15.2)
30 days	13 (6.7)	10 (3.4)	24 (9.1)	4 (12.1)
in-hospital	10 (5.1)	6 (2.1)	17 (6.5)	3 (9.1)
AKI	5 (4.7)	6 (3.8)	18 (10.5)	6 (22.2)

Data are reported as number of patients (percentage); P-value < 0.05 = *, p value < 0.01 = **, all values are compared with eGFR ≥90mL/min group. eGFR estimated glomerular filtration rate; MACCE, major adverse cardiac/cerebrovascular events; BARC, Bleeding Academic Research Consortium; BARC 1, minimal bleeding complication, BARC 2, requiring nonsurgical, medical intervention by a healthcare professional, leading to hospitalization or increased level of care, or prompting evaluation, but not fit to criteria of BARC>2, Major bleeding complication including fatal bleeding Data are reported as number and percentage or mean ± SD; TIA, transient ischemic attack, AKI = Acute kidney injure with >26.5 μmol/l increase of creatinine.

**Table 5:** Univariate Cox regression and multivariate nested adjusted models on the relative risk (hazard ratio) of renal impairment on all-cause mortality; major adverse cardiac and cerebrovascular events; and clinically significant bleeding (BARC 2, 3, 5) in patients with AF undergoing PCI as compared with normal eGFR (>90ml/kg/min).

	Univariate	Model A	Model B	Model C
All-cause mortality	2.22 (1.70–2.89)	2.08 (1.54–2.80)	1.99 (1.49–2.67)	2.04 (1.51–2.75)
MACCE	1.61 (1.33–1.93)	1.51 (1.21–1.88)	1.49 (1.20–1.85)	1.51 (1.21–1.89)
BARC 2,3 and 5	1.26 (1.04–1.52)	1.09 (0.86–1.37)	1.08 (0.86–1.36)	1.08 (0.85–1.37)

Model A (age (as a continuous variable), sex)

Model B (age, sex, acute coronary syndrome)

Model C (age, sex, acute coronary syndrome, previous myocardial infarction, congestive heart failure, center)

Patients with severe renal impairment had a very high risk of all-cause mortality (HR 13.6 95%Cl 5.77–32.1 p<0.001) with 45% total mortality. Also the risk of MACCE (HR 6.00 95%CI 3.31–10.9, p<0.001) was increased compared to those with normal renal function.

### Renal impairment after PCI

Post-procedural creatinine level was available in 465 (59.5%) of the 781 patients. Acute kidney injury occurred in 35 (7.5%) of 465 patients after PCI affecting 4.7% of patients with normal renal function; 3.8% with mild; 10.5% with moderate; and 22.2% with severe renal impairment, respectively.

In a Cox regression model adjusting for age as a continuous variable, gender, pre-PCI eGFR and acute coronary syndrome as an indication for PCI, acute kidney injury was an independent predictor of all-cause mortality (HR 2.32, 95%CI 1.15–4.68, p = 0.02) and MACCE (HR 1.90, 95%CI 1.06–3.38, p = 0.03) with a trend to effect on bleeding events (HR 1.62, 95%Cl 0.96–2.76, p = 0.07) compared to patients with no significant impairment in renal function.

## Discussion

Renal impairment and AF are two well-known independent high-risk features for complications in patients undergoing PCI. In this prospective multicenter real-world registry, the outcomes of these patients with both of these risk factors—often excluded from clinical trials—were analyzed for the first time. We showed that 75% of these patients have CKD according to the current criteria. Importantly, one third of the patients had mild renal impairment (eGFR 60-89mL/min) often unrecognized because the creatinine levels typically lie within the normal range. The principal novel finding is that even this early renal impairment is clinically important and results in an increase in mortality and MACCE.

Although the major impact of renal impairment on the outcome following PCI has long been recognized, less is known about the impact of mild renal impairment. Compared to those with normal renal function, the effect of mild renal impairment on survival and major adverse cardiac event rate was recently reported to be negligible in the general PCI population [27]. However, it was a significant predictor of worse prognosis in patients presenting with ST-elevation myocardial infarction [28]. In addition, Reinecke et al. reported that mildly elevated creatinine levels were associated with a twofold increase in total mortality and a 10% reduction in cumulative survival over three years in the general PCI population [29]. A similar phenomenon was observed by Gibson et al. who found that eGFR < 90 mL/min remained independently associated with increased mortality in non-ST-segment elevation ACS patients [30].

Cardiovascular events are the most common cause of death in patients with renal impairment. Explanations of this interaction include the greater frequency of risk factors, such as hypertension and diabetes mellitus. In addition, factors associated with renal disease such as uremic toxins, inflammation, hyperparathyroidism, elevated calcium-phosphate product, fluid overload, and anemia contribute to the severity and extent of the coronary atherosclerosis as well as the higher adverse event rates in this high risk population. Adverse events may also be related to other conditions than epicardial coronary artery disease such as uremic cardiomyopathy, metabolic derangements, and microvascular disease. Nevertheless, the presence of significant coronary artery stenosis has been reported to worsen the prognosis dramatically [27].

Reflecting this background, patients in the AFCAS study with severe renal insufficiency had more often high-risk baseline characteristics such as multivessel and left main disease and underwent more often emergency PCI due to acute coronary syndrome. In our study, patients with severe renal impairment had an extremely poor prognosis with almost 50% mortality within 12 months.

A dose-dependent effect of worsening renal function was observed on thrombotic and bleeding events in this high-risk patient group. In an earlier smaller study, Manzano-Fernades et al. reported nearly 2.5-fold increase in the risk of PCI associated major bleeding in patients with mild renal impairment and AF [14]. Our study extends these findings showing that decreased eGFR values were associated with an increased risk of any BARC bleeding in patients with AF undergoing PCI, but only a trend in clinically significant bleeding events. In multivariate analysis, patients with mild stage of CKD had a non-significant trend to increased bleeding events, whereas patients with moderate and severe renal impairment had clearly elevated risks. Of clinical interest, thrombotic and thromboembolic events were more common than clinically significant bleeding events suggesting that the prothrombotic risk outweighs the bleeding risk in most patients. Clinical implication of this finding is that potent antithrombotic therapy after PCI should be considered also in patients with impaired renal function without overt fear for bleeding complications.

Acute kidney injury is a frequent complication of PCI especially in patients with acute coronary syndrome and seems to be associated with increased risk of in-hospital morbidity and mortality [31]. In line with this experience, acute kidney injury occurred in a total of 7.5% after PCI and was an independent predictor of mortality and MACCE in the present study emphasizing the importance of its prevention with adequate hydration and restricted use of contrast agent.

Repeat revascularization rates were relatively low in all eGFR groups when compared to previous studies and especially those evaluating restenosis by repeat angiography [32]. This might be explained by the older age and high prevalence of co-morbidities including AF, which might have favoured medical treatment over re-intervention in patients with recurrent angina after PCI. Moreover, no angiographic follow-up was performed, and all target vessel revascularizations procedures were ischemia-driven. It is known that ischemia-driven target vessel revascularizations tend to underestimate the actual rates of restenosis. In addition, the absence of symptoms of restenosis in patients with renal impairment may lead to silent ischemia and contribute to the high risk of subsequent cardiac events. The rate of stent thrombosis was relatively low in all eGFR groups, and the low number of events precludes comparison between the groups.

## Limitations

This study has all the inherent limitations of an observational study including individual risk based decision-making in the treatment choices. This study was planned before CKD-EPI formula was adopted in general use and renal function was evaluated using the Cockcroft-Gault formula. The choice of stent and antithrombotic treatment has been entirely at the treating physician’s discretion. Moreover, multivariate models will not fully cover potential residual confounding. Despite these limitations we believe that these data are of value in guiding the treatment of patients with AF undergoing PCI. The strength of our study is the inclusion of ‘real-world’ PCI patients in the largest dataset so far also including a substantial number of patients with acute coronary syndrome. Bleeding outcomes were defined according to the latest BARC definition [26].

## Conclusions

Renal impairment is a frequent finding in patients with AF undergoing PCI and only one fourth of the patients had normal renal function. Even mild renal impairment was associated with an increase in all-cause mortality and MACCE with a trend towards increased bleeding events. Patients with severe renal dysfunction and AF have a very poor prognosis with high MACCE rate and mortality within the first year after PCI. Also, patients with acute kidney injury carry an elevated risk, which might potentially be avoidable with preventive measures.

## References

[1] Jha V., Garcia-Garcia G., Iseki K., Li Z., Naicker S., Plattner B., et al., "Chronic kidney disease: global dimension and perspectives," *Lancet*, vol. 382, pp. 260–72, 7 2013 10.1016/S0140-6736(13)60687-X 23727169

[2] Soliman E. Z., Prineas R. J., Go A. S., Xie D., Lash J. P., Rahman M., et al., "Chronic kidney disease and prevalent atrial fibrillation: the Chronic Renal Insufficiency Cohort (CRIC)," *Am Heart J*, vol. 159, pp. 1102–7, 6 2010 10.1016/j.ahj.2010.03.027 20569726 PMC2891979

[3] Marinigh R., Lane D. A., and Lip G. Y., "Severe renal impairment and stroke prevention in atrial fibrillation: implications for thromboprophylaxis and bleeding risk," *J Am Coll Cardiol*, vol. 57, pp. 1339–48, 3 2011 10.1016/j.jacc.2010.12.013 21414530

[4] Baber U., Howard V. J., Halperin J. L., Soliman E. Z., Zhang X., McClellan W., et al., "Association of chronic kidney disease with atrial fibrillation among adults in the United States: REasons for Geographic and Racial Differences in Stroke (REGARDS) Study," *Circ Arrhythm Electrophysiol*, vol. 4, pp. 26–32, 2 2011 10.1161/CIRCEP.110.957100 21076159 PMC3049935

[5] Nelson S. E., Shroff G. R., Li S., and Herzog C. A., "Impact of chronic kidney disease on risk of incident atrial fibrillation and subsequent survival in medicare patients," *J Am Heart Assoc*, vol. 1, p. e002097, 8 2012 10.1161/JAHA.112.002097 23130165 PMC3487349

[6] Go A. S., Chertow G. M., Fan D., McCulloch C. E., and Hsu C. Y., "Chronic kidney disease and the risks of death, cardiovascular events, and hospitalization," *N Engl J Med*, vol. 351, pp. 1296–305, 9 2004 15385656 10.1056/NEJMoa041031

[7] Barsoum R. S., "Chronic kidney disease in the developing world," *N Engl J Med*, vol. 354, pp. 997–9, 3 2006 16525136 10.1056/NEJMp058318

[8] Iguchi Y., Kimura K., Kobayashi K., Aoki J., Terasawa Y., Sakai K., et al., "Relation of atrial fibrillation to glomerular filtration rate," *Am J Cardiol*, vol. 102, pp. 1056–9, 10 2008 10.1016/j.amjcard.2008.06.018 18929708

[9] Alonso A., Lopez F. L., Matsushita K., Loehr L. R., Agarwal S. K., Chen L. Y., et al., "Chronic kidney disease is associated with the incidence of atrial fibrillation: the Atherosclerosis Risk in Communities (ARIC) study," *Circulation*, vol. 123, pp. 2946–53, 6 2011 10.1161/CIRCULATIONAHA.111.020982 21646496 PMC3139978

[10] Kaya E., Cuneo A., Hochadel M., Jünger C., Stepper W., Bramlage P., et al., "Impact of chronic kidney disease on the prognosis of patients undergoing percutaneous coronary interventions using drug-eluting stents," *Clin Res Cardiol*, vol. 100, pp. 1103–9, 12 2011 10.1007/s00392-011-0347-7 21912915

[11] Lambert N. D., Sacrinty M. T., Ketch T. R., Turner S. J., Santos R. M., Daniel K. R., et al., "Chronic kidney disease and dipstick proteinuria are risk factors for stent thrombosis in patients with myocardial infarction," *Am Heart J*, vol. 157, pp. 688–94, 4 2009 10.1016/j.ahj.2009.01.009 19332197

[12] Latif F., Kleiman N. S., Cohen D. J., Pencina M. J., Yen C. H., Cutlip D. E., et al., "In-hospital and 1-year outcomes among percutaneous coronary intervention patients with chronic kidney disease in the era of drug-eluting stents: a report from the EVENT (Evaluation of Drug Eluting Stents and Ischemic Events) registry," *JACC Cardiovasc Interv*, vol. 2, pp. 37–45, 1 2009 10.1016/j.jcin.2008.06.012 19463396

[13] Parikh P. B., Jeremias A., Naidu S. S., Brener S. J., Lima F., Shlofmitz R. A., et al., "Impact of severity of renal dysfunction on determinants of in-hospital mortality among patients undergoing percutaneous coronary intervention," *Catheter Cardiovasc Interv*, vol. 80, pp. 352–7, 9 2012 10.1002/ccd.23394 22566286

[14] Manzano-Fernández S., Cambronero F., Caro-Martínez C., Hurtado-Martínez J. A., Marín F., Pastor-Pérez F. J., et al., "Mild kidney disease as a risk factor for major bleeding in patients with atrial fibrillation undergoing percutaneous coronary stenting," *Thromb Haemost*, vol. 107, pp. 51–8, 1 2012 10.1160/TH11-08-0524 22072287

[15] Levey A. S., Coresh J., Balk E., Kausz A. T., Levin A., Steffes M. W., et al., "National Kidney Foundation practice guidelines for chronic kidney disease: evaluation, classification, and stratification," *Ann Intern Med*, vol. 139, pp. 137–47, 7 2003 12859163 10.7326/0003-4819-139-2-200307150-00013

[16] Foundation N. K., "K/DOQI clinical practice guidelines for chronic kidney disease: evaluation, classification, and stratification," *Am J Kidney Dis*, vol. 39, pp. S1–266, 2 2002 11904577

[17] Lip G. Y., Huber K., Andreotti F., Arnesen H., Airaksinen J. K., Cuisset T., et al., "Antithrombotic management of atrial fibrillation patients presenting with acute coronary syndrome and/or undergoing coronary stenting: executive summary—a Consensus Document of the European Society of Cardiology Working Group on Thrombosis, endorsed by the European Heart Rhythm Association (EHRA) and the European Association of Percutaneous Cardiovascular Interventions (EAPCI)," *Eur Heart J*, vol. 31, pp. 1311–8, 6 2010 10.1093/eurheartj/ehq117 20447945

[18] Rubboli A., Halperin J. L., Airaksinen K. E., Buerke M., Eeckhout E., Freedman S. B., et al., "Antithrombotic therapy in patients treated with oral anticoagulation undergoing coronary artery stenting. An expert consensus document with focus on atrial fibrillation," *Ann Med*, vol. 40, pp. 428–36, 2008 10.1080/07853890802089786 18608125

[19] Olesen J. B., Lip G. Y., Kamper A. L., Hommel K., Køber L., Lane D. A., et al., "Stroke and bleeding in atrial fibrillation with chronic kidney disease," *N Engl J Med*, vol. 367, pp. 625–35, 8 2012 10.1056/NEJMoa1105594 22894575

[20] Apostolakis S., Guo Y., Lane D. A., Buller H., and Lip G. Y., "Renal function and outcomes in anticoagulated patients with non-valvular atrial fibrillation: the AMADEUS trial," *Eur Heart J*, 8 2013. 10.1093/eurheartj/eht328 23966309

[21] Lahtela H., Rubboli A., Schlitt A., Karjalainen P. P., Niemelä M., Vikman S., et al., "Heparin bridging vs. uninterrupted oral anticoagulation in patients with Atrial Fibrillation undergoing Coronary Artery Stenting. Results from the AFCAS registry," *Circ J*, vol. 76, pp. 1363–8, 2012 22447005 10.1253/circj.cj-11-1206

[22] Schlitt A., Rubboli A., Lip G. Y., Lahtela H., Valencia J., Karjalainen P. P., et al., "The management of patients with atrial fibrillation undergoing percutaneous coronary intervention with stent implantation: In-hospital-data from the atrial fibrillation undergoing coronary artery stenting study," *Catheter Cardiovasc Interv*, 6 2013. 10.1002/ccd.25064 23765437

[23] Kiviniemi T., Karjalainen P., Rubboli A., Schlitt A., Tuomainen P., Niemelä M., et al., "Thrombocytopenia in patients with atrial fibrillation on oral anticoagulation undergoing percutaneous coronary intervention," *Am J Cardiol*, vol. 112, pp. 493–8, 8 2013 10.1016/j.amjcard.2013.04.007 23672991

[24] Puurunen M., Kiviniemi T., Nammas W., Schlitt A., Rubboli A., Nyman K., et al., "Impact of anaemia on clinical outcome in patients with atrial fibrillation undergoing percutaneous coronary intervention: insights from the AFCAS registry," *BMJ Open*, vol. 4, p. e004700, 2014 10.1136/bmjopen-2013-004700 24823675 PMC4025460

[25] Thygesen K., Alpert J. S., Jaffe A. S., Simoons M. L., Chaitman B. R., White H. D., et al., "Third universal definition of myocardial infarction," *Circulation*, vol. 126, pp. 2020–35, 10 2012 10.1161/CIR.0b013e31826e1058 25689940

[26] Mehran R., Rao S. V., Bhatt D. L., Gibson C. M., Caixeta A., Eikelboom J., et al., "Standardized bleeding definitions for cardiovascular clinical trials: a consensus report from the Bleeding Academic Research Consortium," *Circulation*, vol. 123, pp. 2736–47, 6 2011 10.1161/CIRCULATIONAHA.110.009449 21670242

[27] Simsek C., Magro M., Boersma E., Onuma Y., Nauta S., Valstar G., et al., "Impact of renal insufficiency on safety and efficacy of drug-eluting stents compared to bare-metal stents at 6 years," *Catheter Cardiovasc Interv*, vol. 80, pp. 18–26, 7 2012 10.1002/ccd.23199 21735520

[28] Campbell N. G., Varagunam M., Sawhney V., Ahuja K. R., Salahuddin N., De Palma R., et al., "Mild chronic kidney disease is an independent predictor of long-term mortality after emergency angiography and primary percutaneous intervention in patients with ST-elevation myocardial infarction," *Heart*, vol. 98, pp. 42–7, 1 2012. 21880649 10.1136/heartjnl-2011-300024

[29] Reinecke H., Trey T., Matzkies F., Fobker M., Breithardt G., and Schaefer R. M., "Grade of chronic renal failure, and acute and long-term outcome after percutaneous coronary interventions," *Kidney Int*, vol. 63, pp. 696–701, 2 2003 12631136 10.1046/j.1523-1755.2003.00784.x

[30] Gibson C. M., Dumaine R. L., Gelfand E. V., Murphy S. A., Morrow D. A., Wiviott S. D., et al., "Association of glomerular filtration rate on presentation with subsequent mortality in non-ST-segment elevation acute coronary syndrome; observations in 13,307 patients in five TIMI trials," *Eur Heart J*, vol. 25, pp. 1998–2005, 11 2004. 15541835 10.1016/j.ehj.2004.08.016

[31] Marenzi G., Cabiati A., Bertoli S. V., Assanelli E., Marana I., De Metrio M., et al., "Incidence and relevance of acute kidney injury in patients hospitalized with acute coronary syndromes," *Am J Cardiol*, vol. 111, pp. 816–22, 3 2013 10.1016/j.amjcard.2012.11.046 23273525

[32] Schoebel F. C., Gradaus F., Ivens K., Heering P., Jax T. W., Grabensee B., et al., "Restenosis after elective coronary balloon angioplasty in patients with end stage renal disease: a case-control study using quantitative coronary angiography," *Heart*, vol. 78, pp. 337–42, 10 1997. 9404246 10.1136/hrt.78.4.337 PMC1892250

